# Inverse Modulation of Aurora Kinase A and Topoisomerase IIα in Normal and Tumor Breast Cells upon Knockdown of Mitochondrial ASncmtRNA

**DOI:** 10.3390/ncrna9050059

**Published:** 2023-10-02

**Authors:** Maximiliano F. Bendek, Christopher Fitzpatrick, Emanuel Jeldes, Anne Boland, Jean-François Deleuze, Nicole Farfán, Jaime Villegas, Gino Nardocci, Martín Montecino, Luis O. Burzio, Verónica A. Burzio

**Affiliations:** 1 Centers of Research Excellence in Science and Technology, Science & Life, Santiago 8580702, Chile; maxbendek@gmail.com (M.F.B.); christopher-shawn.fitzpatrick@inrae.fr (C.F.);; 2 Unit of Molecular Virology and Immunology, INRAE, University of Paris-Saclay, 78350 Jouy-en-Josas, France; 3 Beatson Institute for Cancer Research, Glasgow G61 1BD, UK; 4 CEA, National Center for Research in Human Genomics (NCRHG), University of Paris-Saclay, 91057 Evry, France; 5 Department of Biological Sciences, Faculty of Life Sciences, University of Andrés Bello, Santiago 8370146, Chile; lburzio@gmail.com; 6 Faculty of Health and Social Sciences, University of Las Americas, Santiago 8242125, Chile; 7 School of Veterinary Medicine, Faculty of Life Sciences, University of Andrés Bello, Santiago 8370146, Chile; 8 Center of Interventional Medicine for Precision and Advanced Cellular Therapy, Faculty of Medicine, University of Los Andes, Santiago 7620086, Chile; 9 Center for Biomedical Research and Innovation (CIIB), Faculty of Medicine, University of Los Andes, Santiago 7620086, Chile; 10 Institute of Biomedical Sciences, Faculty of Medicine and Faculty of Life Sciences, University of Andrés Bello, Santiago 8370146, Chile

**Keywords:** ncRNA, Aurora kinase A, toposiomerase IIα, breast cancer, antisense therapy

## Abstract

Breast cancer is currently the most diagnosed form of cancer and the leading cause of death by cancer among females worldwide. We described the family of long non-coding mitochondrial RNAs (ncmtRNAs), comprised of sense (SncmtRNA) and antisense (ASncmtRNA) members. Knockdown of ASncmtRNAs using antisense oligonucleotides (ASOs) induces proliferative arrest and apoptotic death of tumor cells, but not normal cells, from various tissue origins. In order to study the mechanisms underlying this selectivity, in this study we performed RNAseq in MDA-MB-231 breast cancer cells transfected with ASncmtRNA-specific ASO or control-ASO, or left untransfected. Bioinformatic analysis yielded several differentially expressed cell-cycle-related genes, from which we selected Aurora kinase A (*AURKA*) and topoisomerase IIα (*TOP2A*) for RT-qPCR and western blot validation in MDA-MB-231 and MCF7 breast cancer cells, as well as normal breast epithelial cells (HMEC). We observed no clear differences regarding mRNA levels but both proteins were downregulated in tumor cells and upregulated in normal cells. Since these proteins play a role in genomic integrity, this inverse effect of ASncmtRNA knockdown could account for tumor cell downfall whilst protecting normal cells, suggesting this approach could be used for genomic protection under cancer treatment regimens or other scenarios.

## 1. Introduction

Breast cancer (BrCa) is currently the most diagnosed form of cancer among both sexes and the leading cause of cancer death in females worldwide [1]. The development of effective treatments against this malignancy is hindered by its largely heterogeneous nature and BrCa is thus classified into three subtypes, of which the triple-negative subtype is the most aggressive and most difficult to treat [2]. Front-line BrCa treatments include drugs that disturb the correct segregation of sister chromatids during mitosis, which are not specific for cancer cells and exert side effects that hinder quality of life [2].

The underlying molecular causes of BrCa include genetic and epigenetic causes, among which are alterations in expression levels of non-coding RNAs (ncRNAs) [3]. We previously described the family of long non-coding mitochondrial RNAs (ncmtRNAs), originating from the mitochondrial 16S rRNA gene, and comprised of sense (SncmtRNA) and antisense (ASncmtRNA-1 and -2) members [4,5,6]. Knockdown (KD) of ASncmtRNAs with an antisense oligonucleotide (ASO-Andes-1537) triggers proliferative arrest and apoptotic death in tumor cells [7,8,9,10,11,12] without affecting normal cell viability [7,8,9,10]. In in vivo mouse models of different murine and human cancers, including breast cancer, ASncmtRNA KD precludes tumor growth and metastasis [8,9,10,11,12]. In MDA-MB-231 triple-negative breast cancer cells, these effects are underlain by an arrest in the S-phase and downregulation of the key cell cycle progression factors cyclin B1, cyclin D1, CDK1, CDK4, and survivin [12]. The latter not only acts as a cell cycle progression factor during the M-phase, but is also an important member of the inhibitor of apoptosis (IAP) family of proteins and, thus, its downfall could be key to the massive induction of apoptosis observed in several tumor cell lines after ASncmtRNA KD [7,8,9,10]. Of note, KD of SncmtRNA does not exert noticeable effects on tumor or normal cells (unpublished).

In the present study, we performed a transcriptome analysis on MDA-MB-231 cells after ASncmtRNA KD, in order to gain a deeper understanding on the mechanisms behind the selectivity of this approach against tumor cells. In the bioinformatic analysis of our data, we found additional differentially modulated cell-cycle-related genes, among which Aurora kinase A (*AURKA*) and topoisomerase IIα (*TOP2A*) stood out to us, since both are essential in ensuring the correct segregation of sister chromatids during mitosis, thereby protecting genomic integrity during mitotic cell division [13,14,15,16,17]. We validated these changes in MDA-MB-231 and MCF7 breast cancer cell lines, in order to determine which are common to two different breast cancer subtypes. We also analyzed normal human mammary epithelial cells (HMEC) to establish differential events between tumor and normal cells. *AURKA* and *TOP2A* were downregulated in both tumor cell lines but were, conversely, strongly upregulated in HMECs. This inverse behavior could explain the selectivity of the treatment against tumor cells [7,8,9,10].

## 2. Results

### Transcriptomic Analysis of MDA-MB-231 Breast Cancer Cells after AsncmtRNA KD

**Figure 1:**
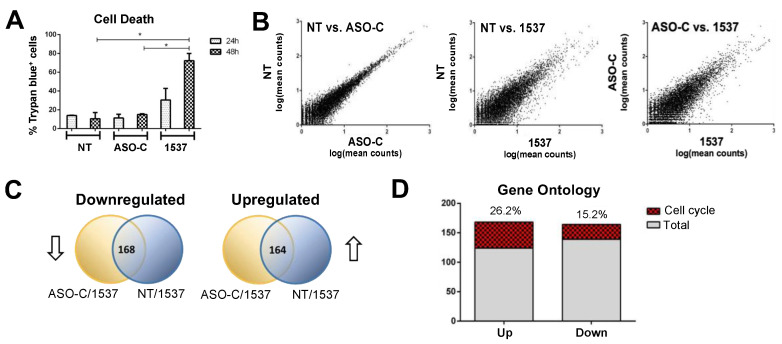
Transcriptomic analysis of MDA-MB-231 cells after knockdown of ASncmtRNA. Cells were transfected with 200 nM ASO-C or Andes-1537 or left untreated. (**A**) A representative experiment showing cell death measured by Trypan blue incorporation at 24 and 48 h. Andes-1537-treated cells displayed significantly higher % of death at 48 h, compared to both controls. The RNA samples used for sequencing were extracted from cells treated in this manner for 24 h in order to detect early events. * *p* < 0.05. (**B**) Dispersion plots showing a comparison between all conditions of total gene expression expressed in TPM. (**C**) Venn diagrams showing common downregulated (left) and upregulated (right) genes between NT vs. 1537 and ASO-C vs. 1537. (**D**) Gene ontology analysis revealed that an important proportion of differentially expressed genes were cell cycle related.

To identify additional cell cycle genes differentially expressed by KD of ASncmtRNAs, we performed ASncmtRNA KD in MDA-MB-231 cells transfected with Andes-1537 and, as negative controls, we transfected cells with a non-related ASO (control ASO or ASO-C) or left them untreated (non-transfected, NT) (Materials and Methods). We corroborated, as observed previously [12], 30% and 70% cell death at 24 and 48 h, respectively, compared to 10% in the controls (Figure 1A). Since the degree of cell death observed at 48 h should be the result of earlier events, including changes in gene expression, we investigated mRNA levels at 24 h post-transfection using RNA sequencing and bioinformatic analysis. A comparison between both controls, non-treated (NT), and control ASO (ASO-C)-transfected cells, showed a high correlation of differentially expressed genes (Figure 1B). In contrast, both controls showed higher dispersion in a similar fashion when compared to Andes-1537-transfected cells (Figure 1B). This behavior can also be seen in volcano plots of Andes-1537 compared to each control (Supplementary Figure S1A,B). As shown in Figure 1C, 168 genes were downregulated comparing both NT and ASO-C controls to Andes-1537, and 164 genes were upregulated, a high proportion of which corresponded to cell cycle genes (Figure 1D). Among these gene classifications, some of the biological function categories were related to chromosomal integrity maintenance, such as chromosome segregation, centrosome cycle, and centrosome assembly (Supplementary Figure S1C,D, red asterisks). Gene ontology analysis on the DAVID platform resulted in several gene categories directly related to cell cycle progression that were downregulated compared to controls (Supplementary Figure S1E). Interestingly, upregulated gene categories included cell cycle arrest and cellular response to DNA damage stimulus (Supplementary Figure S1F).

Using the CycleBase platform, we generated a list of pre-selected cell-cycle-related genes for RT-qPCR validation (Supplementary Figure S2A). In order to define the final genes for western blot analysis, we first performed RT-qPCR on all three cell lines (tumor lines MDA-MB-231 and MCF7, and normal HMEC cells). Our primary aim was to study only genes that exhibited similar behavior in both tumor lines, in order to establish potentially universal primary triggers for the detrimental effects we have observed in tumor cells from a wide array of tumor cell lines [7,8,9,10,11,12]. Of this list of 14 downregulated and 5 upregulated genes, only 8 genes fulfilled this requirement, from which we chose the final three to further analyze for the present study. First, we selected *CCNB1* as an internal control to validate our transcriptomic approach, since we had previously observed that the mRNA of this gene was downregulated in MDA-MB-231 cells (unpublished) and protein level was reduced after the ASncmtRNA KD treatment [12]. The other genes selected for this study were *AURKA* and *TOP2A*, merely because we became highly interested when we encountered these two genes that are involved in the maintenance of genome integrity during mitotic cell division and not only as “classic” cell cycle progression factors. Supplementary Figure S2B depicts the closest interactions of each of these three genes where, interestingly, some of the members of the list of pre-selected genes are involved.

As mentioned above, Cyclin B1 (*CCNB1*) was chosen for detailed analysis as a control for validating our bioinformatic approach. *CCNB1* TPM (transcripts per million) count was reduced by 3.5–4-fold in the bioinformatic analysis in Andes-1537-transfected cells, compared to the controls. RT-qPCR validation in the MDA-MB-231, MCF7, and HMEC cells showed a similar behavior in all three cell types, albeit to a lesser extent (Supplementary Figure S3B). Protein levels showed, as expected, a strong reduction in both tumor cell lines, while there was only a modest decrease in HMECs (Supplementary Figure S3C), reflecting the lack of a strong effect of the KD treatment on normal cells.

**Figure 2:**
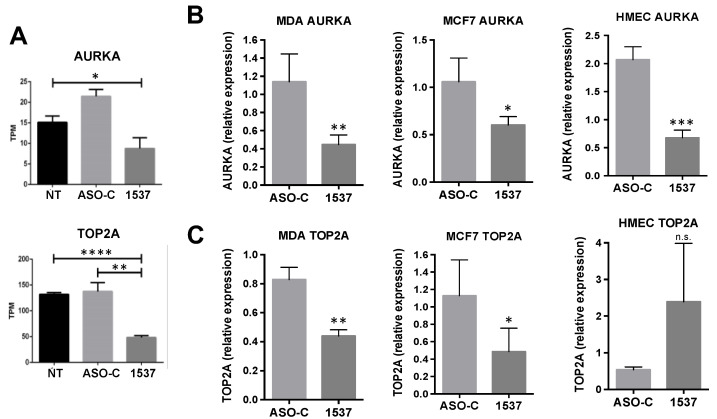
Relative expression of *AURKA* and *TOP2A* mRNA upon ASncmtRNA KD. (**A**) Relative abundance of mRNAs in the MDA-MB-231 transcriptome, expressed as transcripts per million (TPM), for the three conditions. These results were validated by RT-qPCR in the same cell line, the MCF7 line, and normal primary breast epithelial cells (HMEC), after a 24 h treatment. (**B**) *AURKA* mRNA is downregulated in all three cell types. (**C**) *TOP2A* was significantly downregulated in both tumor cell lines but not in HMECs. In (**B**,**C**), results are plotted relative to NT cells. * *p* < 0.05, ** *p* < 0.01, *** *p* < 0.001, **** *p* < 0.0001, n.s. non-significant.

**Figure 3:**
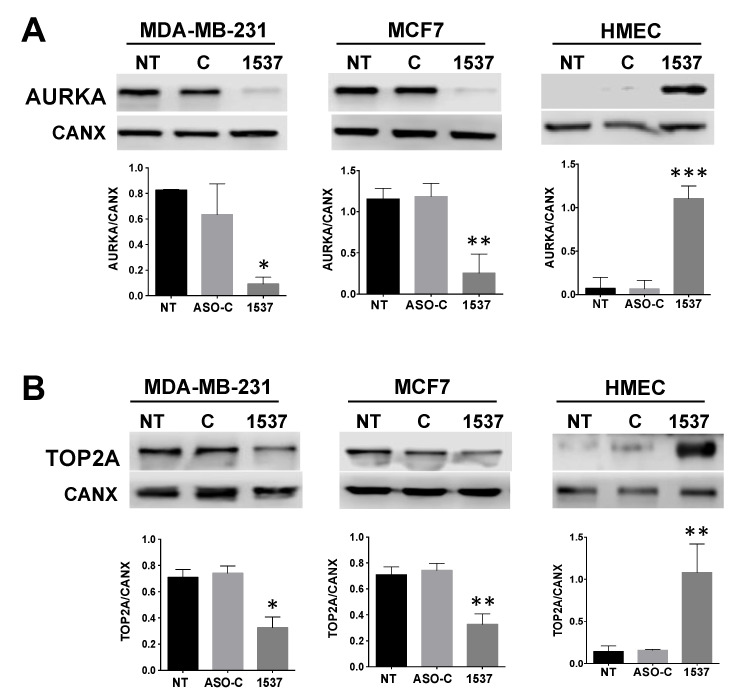
Protein expression of *AURKA* and *TOP2A* after ASncmtRNA KD for 24 h in MDA-MB-231, MCF7, and HMEC cells. (**A**) *AURKA* expression shows a strong downregulation in tumor cells and a strong upregulation in normal HMEC cells transfected with Andes-1537, compared to the controls. (**B**) *TOP2A* protein is reduced by half after ASncmtRNA KD in both tumor cell lines, while it increases by around 7 times in HMECs. * *p* < 0.05, ** *p* < 0.01, *** *p* < 0.001.

However, it is possible that the reduction in cell cycle proteins such as *CCNB1* and others [12] could simply be a consequence of ASncmtRNA KD-induced proliferative blockage [7,8,9,10,12]. Therefore, we searched for genes that could constitute potential primary triggers of proliferative arrest. The two genes on which we based this study, *AURKA* and *TOP2A*, are both involved in securing genomic integrity during cell cycle progression [13,14,15,16,17]. In our bioinformatic analysis, *AURKA* TPM displayed a 2–3-fold reduction in Andes-1537-transfected cells, compared to the controls (Figure 2A), while *TOP2A* showed a ~2.6-fold reduction (Figure 2A). RT-qPCR validation in the three cell types showed a 2–3-fold reduction in *AURKA* mRNA (Figure 2B). However, protein levels were strongly reduced, 6–8-fold, in both tumor cell lines but, conversely, increased 11-fold in HMECs (Figure 3A). *TOP2A*, on the other hand, displayed a ~2-fold reduction in its mRNA levels in both tumor cell lines and no significant change in HMECs (Figure 2C), but protein levels were also inversely modulated, with a 2-fold reduction in both tumor cell lines and a 5-fold increase in HMECs (Figure 3C). These observations show that *AURKA* and *TOP2A* are inversely modulated in breast tumor and normal cells by ASncmtRNA KD, which could explain the different responses of tumor and normal cells to this treatment.

## 3. Discussion

First-line breast cancer treatments include DSB-inducing anthracyclines, such as doxorubicin or epirubicin, which are thought to act through topoisomerase II poisoning [2] and taxanes such as paclitaxel or docetaxel, which disrupt mitotic spindle function, thereby introducing gross genetic errors [2]. These drugs and others are usually combined under different regimes or in conjunction with surgery and/or radiotherapy. Chemo- and radiotherapy aim to trigger tumor cell death by inducing irreparable genetic errors, but these treatments also damage healthy cells, causing side effects that hamper quality of life and, in some cases, secondary cancers [18].

Tumor cells from different origins readily express SncmtRNA, while normal cells express both transcript types [4,5,6,7,8,9,10]. As stated above, ASncmtRNA KD induces apoptotic death of tumor cells from different tissues [7,8,9,10,12], thus this approach could be visualized as a stand-alone treatment for different cancer types, including breast cancer. We hypothesized that ASncmtRNA KD-induced proliferative arrest [7,8,9,10,12] is governed by a reduction in cell cycle progression factors. In this work we chose to perform a transcriptome-wide study of Andes-1537-treated cells, compared to controls, in order to address the mRNA levels, one of the factors governing protein levels. Another important instance of protein level regulation lies in translation control, where miRNAs are important actors, usually blocking mRNA translation. In this context, we previously showed that ASncmtRNA KD induces an increase in miRNAs miR-4485 and miR-1973 [12] and we are currently studying the significance of this effect on the genomic integrity of tumor and normal cells, especially focused on *AURKA* and *TOP2A*.

We show here that cyclin B1 is differentially modulated among tumor (strongly downregulated) and normal (mildly downregulated) breast epithelial cells (Supplementary Figure S3C). Interestingly, we encountered an inverse modulation of *AURKA* and *TOP2A* in tumor and normal breast cells, which could constitute the basis for treatment selectivity [7,8,9,10]. Both factors participate in cell cycle progression, ensuring correct segregation of sister chromatids to daughter cells [13,14] and, therefore, a reduction in the levels of these proteins or in their activity should not only preclude cell cycle progression but will also generate gross genetic errors, ultimately leading to apoptotic cell death.

*AURKA* is essential to centrosome duplication, maturation, and separation at the S and G2 phases, and mitotic entry and spindle formation in the M phase [13]. It is also involved in activating the G2 DNA damage checkpoint induced by DSBs [15]. *AURKA* overexpression is commonly observed in many solid tumors including breast cancers [15], enhancing genomic instability, and is the target of several clinical trials with drugs that inhibit its activity [15].

*TOP2A* performs double-strand cuts in DNA for correct decatenation of sister chromatids towards separation at anaphase. Defects in *TOP2A* activity trigger a G2 decatenation checkpoint that stalls the cell cycle at G2/M, protecting against genomic damage, and inactivation of *TOP2A* leads to abnormal chromosome segregation and genomic instability, linked to tumorigenesis [14]. *TOP2A* is overexpressed in many human cancers including breast cancer, rendering this protein a widely used anti-cancer target [16]. Drugs targeting *TOP2A* activity, including etoposide and doxorubicin, induce double-strand breaks (DSBs), resulting in tumor cell death [17]. However, as in many cancer treatments, normal cells are also affected, resulting in side effects, including the development of secondary cancers and cardiotoxicity [17].

Based on the above, a reduction in the levels of *AURKA* and *TOP2A* induced by ASncmtRNA KD should mimic the effects of drugs that target the activity of these enzymes. Therefore, this molecular effect brought on by the KD treatment could constitute the basis for the induction of the apoptotic death of tumor cells [7,8,9,10,12]. In normal cells, on the other hand, one could ask what happens when these oncogenic proteins are overexpressed; could it result in transformation of normal cells? This is highly unlikely since the overexpression is transient and there is no amplification of the genes coding for these factors. Another pending question is related to the basis for this difference observed between tumor and normal cells. One possibility is the gene expression control exerted by miRNAs, which occurs after mRNA synthesis. As mentioned above, we have studied the changes in miRNA expression upon ASncmtRNA KD [12] and we are currently investigating the significance of this aspect on *AURKA* and *TOP2A* levels.

This is the first report to show an inverse behavior of important cell cycle and genomic protection factors in normal and tumor cells. We are currently studying the behavior of these proteins in other tumor/normal cell cultures in order to determine if these effects are common to different tissue types. We are also performing functional assays to elucidate the biological significance of these changes after ASncmtRNA KD, but the data presented here constitutes a basis for the potential application of ASncmtRNA KD as a coadjuvant treatment complementary to conventional cancer therapies. The opposite behavior of *AURKA* and *TOP2A* in tumor and normal breast cells after ASncmtRNA KD could hypothetically enhance genomic instability in tumor cells on treatment with DNA-damaging drugs or radiotherapy, thereby enhancing the efficacy of treatments (and perhaps allowing for reduced dosage), whilst preserving the genomic integrity of normal cells, and thereby protecting healthy tissue from collateral damage. This approach could open new avenues of BrCa therapies based on ASncmtRNA KD as a coadjuvant to chemo- and/or radiotherapy, while maintaining an agreeable quality of life for patients.

## 4. Materials and Methods

### 4.1. Cell Culture and Transfection

MCF-7, MDA-MB-231, and normal human breast epithelial cells (HMECs) were purchased from ATCC (Manassas, VA, USA) and cultured according to ATCC guidelines. For treatments, 60,000 cells/well were seeded into 12-well plates (Nunc, Thermo-Fisher Scientific, Waltham, MA, USA) and transfected the next day with 200 nM unrelated control ASO (ASO-C: 5′-AGGTGGAGTGGATTGGGG) or Andes-1537 (5′-CACCCACCCAAGAACAGG) and 2 μg/mL Lipofectamine2000 (Thermo-Fisher Scientific), or left untreated (NT). All ASOs contained 100% phosphorothioate internucleosidic bonds (Integrated DNA Technologies, Coralville, IA, USA).

### 4.2. RNA Extraction

RNA was isolated using TRIzol (Thermo-Fisher Scientific). The RNA concentration was determined through spectrometry (EPOCH; Biotek Instruments, Winooski, VT, USA) and the integrity was checked on an Experion system (Bio-Rad, Hercules, CA, USA), using the RNA StdSens analysis kit (Bio-Rad). Selected samples had an RIN value > 0.8.

### 4.3. RNA Sequencing

Library construction, including cDNA synthesis, adapter ligation, amplification, and validation was performed using the TruSeq Stranded Total RNA with Ribo-Zero Human/Mouse/Rat kit (Illumina, San Diego, CA, USA). The MiSeq Reagent Kit v3 (Illumina) was used for paired-end sequencing on a HiSeq 2000 platform (Illumina).

### 4.4. Bioinformatic Analysis

Differential gene expression analysis was performed as described in [19]. Reads (fastq format) were processed with SAMTOOLS (v0.1.19.0) and BOWTIE2 v2.1.0.0 was used for mapping to the GRCh37.p13 reference genome (hg19) (www.ncbi.nlm.nih.gov, accessed on 31 March 2018). The HTSeq v0.9.1 package [20] was used to quantify reads and differential gene expression analysis was performed with DESeq2 v1.14.1, considering *p* < 0.05 and fold-change FC(log2) > 1. Pathway analysis (KEGG, biological process and molecular function) was performed with FIDEA (http://circe.med.uniroma1.it/fidea/, accessed on 25 April 2018) [21] and gene lists were loaded onto the DAVID v6.8 (https://david.ncifcrf.gov/home.jsp, accessed on 30 May 2018) [22,23] and PANTHER (http://www.pantherdb.org/, accessed on 23 June 2018) [24] platforms, where genes related to cell cycle according to KEGG pathways, biological process, and Uniprot functional category, were selected. A list of pre-selected genes was obtained using CycleBase 3.0 (https://cyclebase.org/, accessed on 12 August 2018) [25,26,27] and STRING v10.5 (https://string-db.org/ accessed on 18 September 2018) [28] was used to generate gene interaction maps of the final selected genes (*AURKA*; *TOP2A* and *CCNB1*).

### 4.5. RT-qPCR

RT-qPCR was performed using 200 ng total RNA as described before [12]. Relative mRNA levels were determined using the 2^−ΔΔCt^ method [29] using 18S rRNA, COX5B, and TIMM50 as reference genes. The primer sequences are displayed in Supplementary Table S1.

### 4.6. Western Blot

Western blot was performed as described in [12], using rabbit monoclonal antibodies (Cell Signaling, Danvers, MA, USA) against cyclin B1 (1:500), Aurora kinase A (1:500), topoisomerase IIα (1:500), or calnexin (1:1000) and peroxidase-labeled goat anti-rabbit IgG secondary antibody (1:5000; Calbiochem, San Diego, CA, USA).

### 4.7. Graphs and Statistical Analysis

The data were plotted and analyzed using the Graphpad Prism 6 software. The significance was set at the nominal level of *p* < 0.05.

## Data Availability

The data presented in this study are openly available in the SRA database (https://www.ncbi.nlm.nih.gov/sra accessed on 31 March 2018) under the BioProject ID PRJNA1022094.

## Supplementary Materials

The following supporting information can be downloaded at: https://www.mdpi.com/article/10.3390/ncrna9050059/s1, Table S1: Sequence of primers used for qPCR; Figure S1: Transcriptome data analysis.; Figure S2: Selection of genes of interest; Figure S3: Changes in *CCNB1* expression after ASncmtRNA KD.
