# Supplementary material for: Inverse Modulation of Aurora Kinase A and Topoisomerase IIα in Normal and Tumor Breast Cells upon Knockdown of Mitochondrial ASncmtRNA
Source: Noncoding RNA. 2023 Oct 2;9(5):59. doi: 10.3390/ncrna9050059 (PMC10609868; doi:10.3390/ncrna9050059)
Supplement: Supplementary file 1 [file ncrna-09-00059-s001.zip › ncrna-2528054-supplementary.pdf]

Table S1: Sequence of primers used for qPCR

GENE NAME	Forward/Reverse	Sequence (5’ – 3’)
AURKA	Forward	GTCAAGTCCCCTGTCGGTTC
	Reverse	AGTGAGACCCTCTAGCTGTAAT
TOP2A	Forward	AGTGGAGCTCTCCTAACCGA
	Reverse	CATTTACAGGCTGCAATGGTGA
COX5B	Forward	ATCTGGAGGTGGTGTTCCCA
	Reverse	CCAGACGACGCTGGTATTGT
TIMM50	Forward	CAGATCAGGCCGCAGAGATCG
	Reverse	TATGTCCGGCGCAACTGCT
18S	Forward	GTAACCCGTTGAACCCCATT
	Reverse	CATCCAATCGGTAGTAGCG

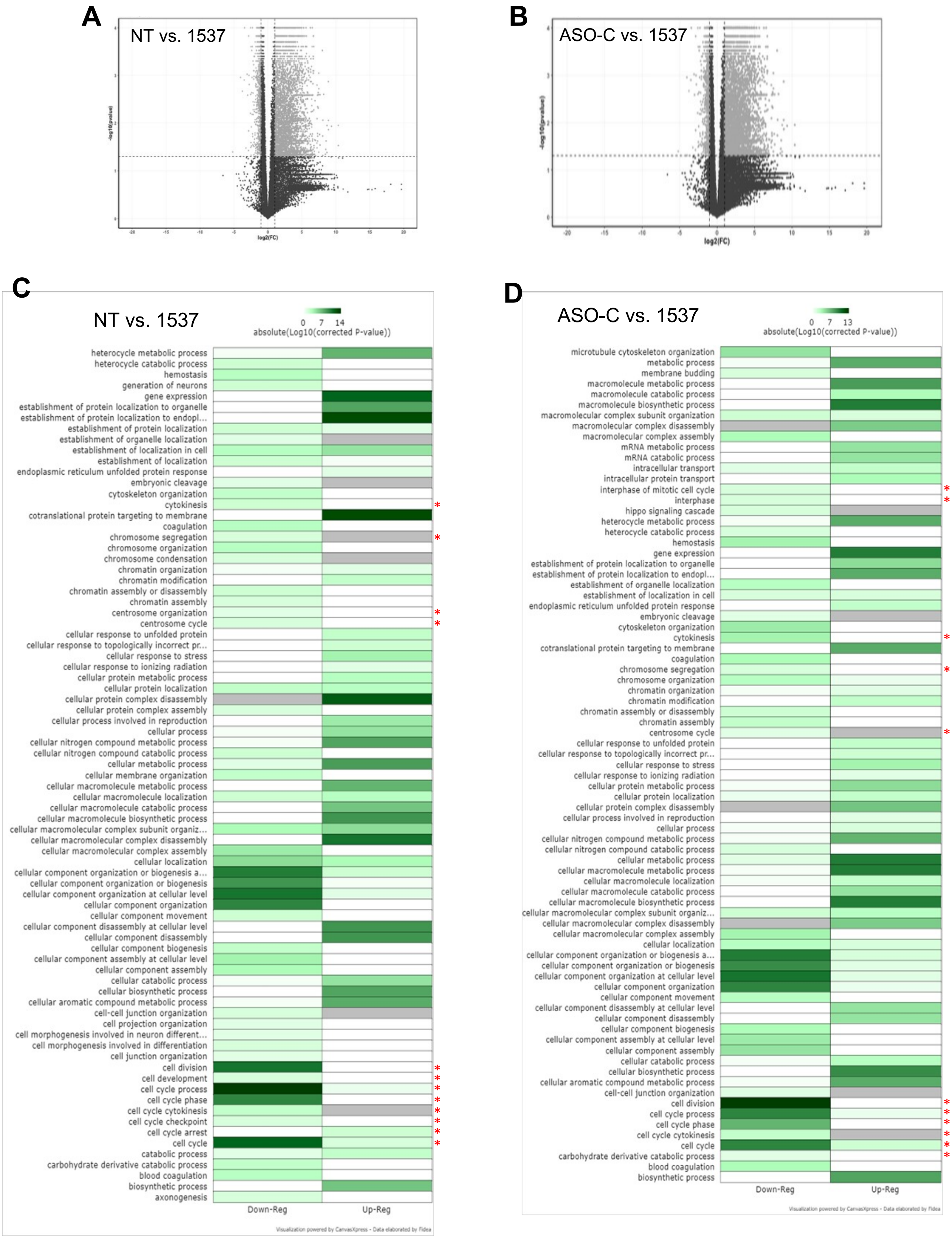

A
NT vs. 1537
-log10(pvalue)
log2(FC)
B
ASO-C vs. 1537
-log10(pvalue)
log2(FC)
C
NT vs. 1537
0 7 14
absolute(Log10(corrected P-value))
heterocycle metabolic process
heterocycle catabolic process
hemostasis
generation of neurons
gene expression
establishment of protein localization to organelle
establishment of protein localization to endopl...
establishment of protein localization
establishment of organelle localization
establishment of localization in cell
establishment of localization
endoplasmic reticulum unfolded protein response
embryonic cleavage
cytoskeleton organization
cytokinesis *
cotranslational protein targeting to membrane
coagulation
chromosome segregation *
chromosome organization
chromosome condensation
chromatin organization
chromatin modification
chromatin assembly or disassembly
chromatin assembly
centrosome organization *
centrosome cycle *
cellular response to unfolded protein
cellular response to topologically incorrect pr...
cellular response to stress
cellular response to ionizing radiation
cellular protein metabolic process
cellular protein localization
cellular protein complex disassembly
cellular protein complex assembly
cellular process involved in reproduction
cellular process
cellular nitrogen compound metabolic process
cellular nitrogen compound catabolic process
cellular metabolic process
cellular membrane organization
cellular macromolecule metabolic process
cellular macromolecule localization
cellular macromolecule catabolic process
cellular macromolecule biosynthetic process
cellular macromolecular complex subunit organiz...
cellular macromolecular complex disassembly
cellular macromolecular complex assembly
cellular localization
cellular component organization or biogenesis a...
cellular component organization or biogenesis
cellular component organization at cellular level
cellular component organization
cellular component movement
cellular component disassembly at cellular level
cellular component disassembly
cellular component biogenesis
cellular component assembly at cellular level
cellular component assembly
cellular catabolic process
cellular biosynthetic process
cellular aromatic compound metabolic process
cell-cell junction organization
cell projection organization
cell morphogenesis involved in neuron different...
cell morphogenesis involved in differentiation
cell junction organization
cell division *
cell development *
cell cycle process *
cell cycle phase *
cell cycle cytokinesis *
cell cycle checkpoint *
cell cycle arrest *
cell cycle *
catabolic process
carbohydrate derivative catabolic process
blood coagulation
biosynthetic process
axonogenesis
Down-Reg
Up-Reg
Visualization powered by CanvasXpress - Data elaborated by Fidea
D
ASO-C vs. 1537
0 7 13
absolute(Log10(corrected P-value))
microtubule cytoskeleton organization
metabolic process
membrane budding
macromolecule metabolic process
macromolecule catabolic process
macromolecule biosynthetic process
macromolecular complex subunit organization
macromolecular complex disassembly
macromolecular complex assembly
mRNA metabolic process
mRNA catabolic process
intracellular transport
intracellular protein transport
interphase of mitotic cell cycle *
interphase *
hippo signaling cascade
heterocycle metabolic process
heterocycle catabolic process
hemostasis
gene expression
establishment of protein localization to organelle
establishment of protein localization to endopl...
establishment of organelle localization
establishment of localization in cell
endoplasmic reticulum unfolded protein response
embryonic cleavage
cytoskeleton organization
cytokinesis *
cotranslational protein targeting to membrane
coagulation
chromosome segregation *
chromosome organization
chromatin organization
chromatin modification
chromatin assembly or disassembly
chromatin assembly
centrosome cycle *
cellular response to unfolded protein
cellular response to topologically incorrect pr...
cellular response to stress
cellular response to ionizing radiation
cellular protein metabolic process
cellular protein localization
cellular protein complex disassembly
cellular process involved in reproduction
cellular process
cellular nitrogen compound metabolic process
cellular nitrogen compound catabolic process
cellular metabolic process
cellular macromolecule metabolic process
cellular macromolecule localization
cellular macromolecule catabolic process
cellular macromolecule biosynthetic process
cellular macromolecular complex subunit organiz...
cellular macromolecular complex disassembly
cellular macromolecular complex assembly
cellular localization
cellular component organization or biogenesis a...
cellular component organization or biogenesis
cellular component organization at cellular level
cellular component organization
cellular component movement
cellular component disassembly at cellular level
cellular component disassembly
cellular component biogenesis
cellular component assembly at cellular level
cellular component assembly
cellular catabolic process
cellular biosynthetic process
cellular aromatic compound metabolic process
cell-cell junction organization
cell division *
cell cycle process *
cell cycle phase *
cell cycle cytokinesis *
cell cycle *
carbohydrate derivative catabolic process *
blood coagulation
biosynthetic process
Down-Reg
Up-Reg
Visualization powered by CanvasXpress - Data elaborated by Fidea

Supplementary Figure S1 (cont.)

E — Downregulated Genes

KEGG Pathways

Term	RT	Genes	Count	%	P-Value	Benjamini
Cell cycle	RT		8	4.9	5.2×10^{-4}	7.8×10^{-2}
Adherens junction	RT		6	3.7	1.3×10^{-3}	9.5×10^{-2}
Viral carcinogenesis	RT		8	4.9	8.9×10^{-3}	3.7×10^{-1}
Pathogenic Escherichia coli infection	RT		4	2.5	2.0×10^{-2}	5.6×10^{-1}
Hippo signaling pathway	RT		6	3.7	2.9×10^{-2}	6.1×10^{-1}
Shigellosis	RT		4	2.5	3.7×10^{-2}	6.3×10^{-1}
Pancreatic cancer	RT		4	2.5	3.8×10^{-2}	5.8×10^{-1}
Oocyte meiosis	RT		5	3.1	3.8×10^{-2}	5.4×10^{-1}
Salmonella infection	RT		4	2.5	7.0×10^{-2}	7.2×10^{-1}
Ubiquitin mediated proteolysis	RT		5	3.1	7.2×10^{-2}	6.9×10^{-1}

Biological Process

Term	RT	Genes	Count	%	P-Value	Benjamini
cell division	RT		17	10.4	9.8×10^{-8}	1.1×10^{-4}
nucleosome assembly	RT		8	4.9	9.6×10^{-5}	5.0×10^{-2}
movement of cell or subcellular component	RT		7	4.3	1.2×10^{-4}	4.2×10^{-2}
viral process	RT		11	6.7	3.6×10^{-4}	9.2×10^{-2}
spindle localization	RT		3	1.8	7.8×10^{-4}	1.5×10^{-1}
positive regulation of fibroblast proliferation	RT		5	3.1	1.4×10^{-3}	2.2×10^{-1}
mitotic nuclear division	RT		9	5.5	1.8×10^{-3}	2.4×10^{-1}
positive regulation by host of viral genome replication	RT		3	1.8	2.1×10^{-3}	2.5×10^{-1}
Golgi organization	RT		5	3.1	4.3×10^{-3}	4.1×10^{-1}
negative regulation of catalytic activity	RT		5	3.1	4.5×10^{-3}	3.9×10^{-1}

Uniprot Functional Category

Term	RT	Genes	Count	%	P-Value	Benjamini
Phosphoprotein	RT		122	74.8	6.1×10^{-21}	1.5×10^{-18}
Acetylation	RT		75	46.0	6.0×10^{-19}	7.3×10^{-17}
Cell cycle	RT		26	16.0	3.0×10^{-11}	2.5×10^{-9}
Cell division	RT		19	11.7	1.2×10^{-9}	7.3×10^{-8}
Cytoplasm	RT		69	42.3	3.5×10^{-8}	1.7×10^{-6}
Mitosis	RT		14	8.6	1.2×10^{-7}	5.0×10^{-6}
Cytoskeleton	RT		28	17.2	1.5×10^{-7}	5.4×10^{-6}
Ubl conjugation	RT		33	20.2	1.8×10^{-6}	5.5×10^{-5}
Nucleus	RT		68	41.7	2.4×10^{-6}	6.6×10^{-5}
Nucleotide-binding	RT		33	20.2	4.9×10^{-6}	1.2×10^{-4}

F — Upregulated Genes

KEGG Pathways

Term	RT	Genes	Count	%	P-Value	Benjamini
Herpes simplex infection	RT		11	6.7	2.3×10^{-5}	3.8×10^{-3}
Epstein-Barr virus infection	RT		9	5.5	4.4×10^{-5}	3.7×10^{-3}
HTLV-I infection	RT		12	7.4	7.4×10^{-5}	4.1×10^{-3}
Chagas disease (American trypanosomiasis)	RT		7	4.3	8.3×10^{-4}	3.4×10^{-2}
Hepatitis B	RT		8	4.9	8.8×10^{-4}	2.9×10^{-2}
Viral carcinogenesis	RT		9	5.5	1.5×10^{-3}	4.1×10^{-2}
ErbB signaling pathway	RT		6	3.7	2.3×10^{-3}	5.4×10^{-2}
HIF-1 signaling pathway	RT		6	3.7	3.6×10^{-3}	7.2×10^{-2}
p53 signaling pathway	RT		5	3.1	5.7×10^{-3}	1.0×10^{-1}
Epithelial cell signaling in Helicobacter pylori infection	RT		5	3.1	5.7×10^{-3}	1.0×10^{-1}

Biological Process

Term	RT	Genes	Count	%	P-Value	Benjamini
PERK-mediated unfolded protein response	RT		5	3.1	2.1×10^{-6}	2.0×10^{-3}
mRNA splicing, via spliceosome	RT		11	6.7	1.5×10^{-5}	7.2×10^{-3}
cell cycle arrest	RT		9	5.5	2.1×10^{-5}	6.8×10^{-3}
antigen processing and presentation of endogenous peptide antigen via MHC class I via ER pathway, TAP-independent	RT		3	1.8	2.0×10^{-4}	4.7×10^{-2}
viral process	RT		10	6.1	8.2×10^{-4}	1.5×10^{-1}
transcription from RNA polymerase II promoter	RT		13	8.0	1.1×10^{-3}	1.6×10^{-1}
response to stress	RT		5	3.1	1.6×10^{-3}	2.0×10^{-1}
cellular response to DNA damage stimulus	RT		8	4.9	1.7×10^{-3}	1.8×10^{-1}
type I interferon signaling pathway	RT		5	3.1	1.9×10^{-3}	1.8×10^{-1}
antigen processing and presentation of exogenous peptide antigen via MHC class I, TAP-independent	RT		3	1.8	2.3×10^{-3}	2.0×10^{-1}

Uniprot Functional Category

Term	RT	Genes	Count	%	P-Value	Benjamini
Phosphoprotein	RT		110	67.5	2.0×10^{-14}	4.0×10^{-12}
Nucleus	RT		77	47.2	2.2×10^{-10}	2.2×10^{-8}
Ubl conjugation	RT		34	20.9	3.8×10^{-7}	2.5×10^{-5}
Acetylation	RT		51	31.3	1.3×10^{-6}	6.3×10^{-5}
Transcription	RT		38	23.3	1.5×10^{-5}	5.9×10^{-4}
Repressor	RT		16	9.8	4.3×10^{-5}	1.4×10^{-3}
Transcription regulation	RT		35	21.5	1.1×10^{-4}	3.2×10^{-3}
DNA-binding	RT		31	19.0	2.9×10^{-4}	7.2×10^{-3}
Biological rhythms	RT		7	4.3	3.7×10^{-4}	8.3×10^{-3}
Activator	RT		15	9.2	4.9×10^{-4}	9.7×10^{-3}

Figure S1: Transcriptome data analysis. **A, B,** volcano plots of data comparison between NT and Andes-1537 (**A**) and between ASO-C and Andes-1537 (**B**) showing *p*-value and fold-change data. **C, D,** *p*-value heatmaps of most significant differentially-expressed genes between NT and Andes-1537 (**C**) and between ASO-C and Andes-1537 (**D**) according to biological function. Categories directly related to cell cycle progression are marked with red asterisks. E, F, Gene ontology analysis of differentially-expressed genes for NT vs Andes-1537 and ASO-C vs Andes-1537 (see Fig. 1C). **E**, downregulated genes; **F**, upregulated genes. The first 10 results for each (Kegg Pathways, biological process and Uniprot functional category) are shown.

Supplementary Figure S2

A

Down-regulated Genes

N°	ENSEMBL_ID	Oficial_name	Description	CB_rank	CB_peak
1	ENSG00000087586	AURKA	aurora kinase A	4	M
2	ENSG00000134690	CDCA8	cell division cycle associated 8	5	G2
3	ENSG00000175063	UBE2C	ubiquitin conjugating enzyme E2 C	8	G2
4	ENSG00000088325	TPX2	TPX2, microtubule nucleation factor	9	M
5	ENSG00000131747	TOP2A	topoisomerase (DNA) II alpha	14	G2
6	ENSG00000169679	BUB1	BUB1 mitotic checkpoint serine/threonine kinase	15	M
7	ENSG00000126787	DLGAP5	DLG associated protein 5	20	M
8	ENSG00000138160	KIF11	kinesin family member 11	30	G2
9	ENSG00000156970	BUB1B	BUB1 mitotic checkpoint serine/threonine kinase B	33	G2/M
10	ENSG00000134057	CCNB1	cyclin B1	49	M
11	ENSG00000136108	CKAP2	cytoskeleton associated protein 2	50	G2/M
12	ENSG00000134222	PSRC1	proline and serine rich coiled-coil 1	81	G2
13	ENSG00000011426	ANLN	anillin actin binding protein	89	M
14	ENSG00000157456	CCNB2	cyclin B2	96	M

Up-regulated Genes

N°	ENSEMBL_ID	Oficial_name	Description	CB_rank	CB_peak
1	ENSG00000177606	JUN	Jun proto-oncogene, AP-1 transcription factor subunit	393	non-periodic
2	ENSG00000175197	DDIT3	DNA damage inducible transcript 3	1568	non-periodic
3	ENSG00000116717	GADD45A	growth arrest and DNA damage inducible alpha	2092	non-periodic
4	ENSG00000165891	E2F7	E2F transcription factor 7	4207	non-periodic
5	ENSG00000125347	IRF1	interferon regulatory factor 1	4357	non-periodic

B

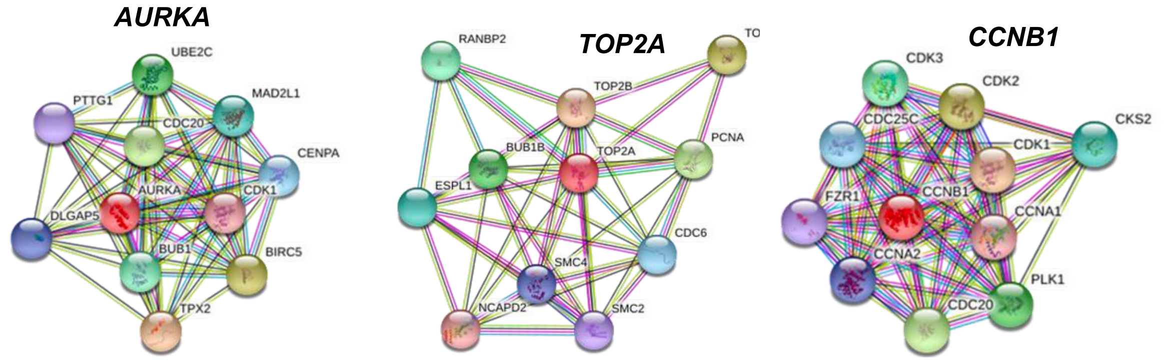

Figure S2: Selection of genes of interest. **A**, final list of pre-selected genes for RT-qPCR validation, generated using CycleBase and selecting only cell cycle-related genes. **B**, Interactions of the final 3 genes selected after RT-qPCR validation, obtained using the STRING platform (see Methods).

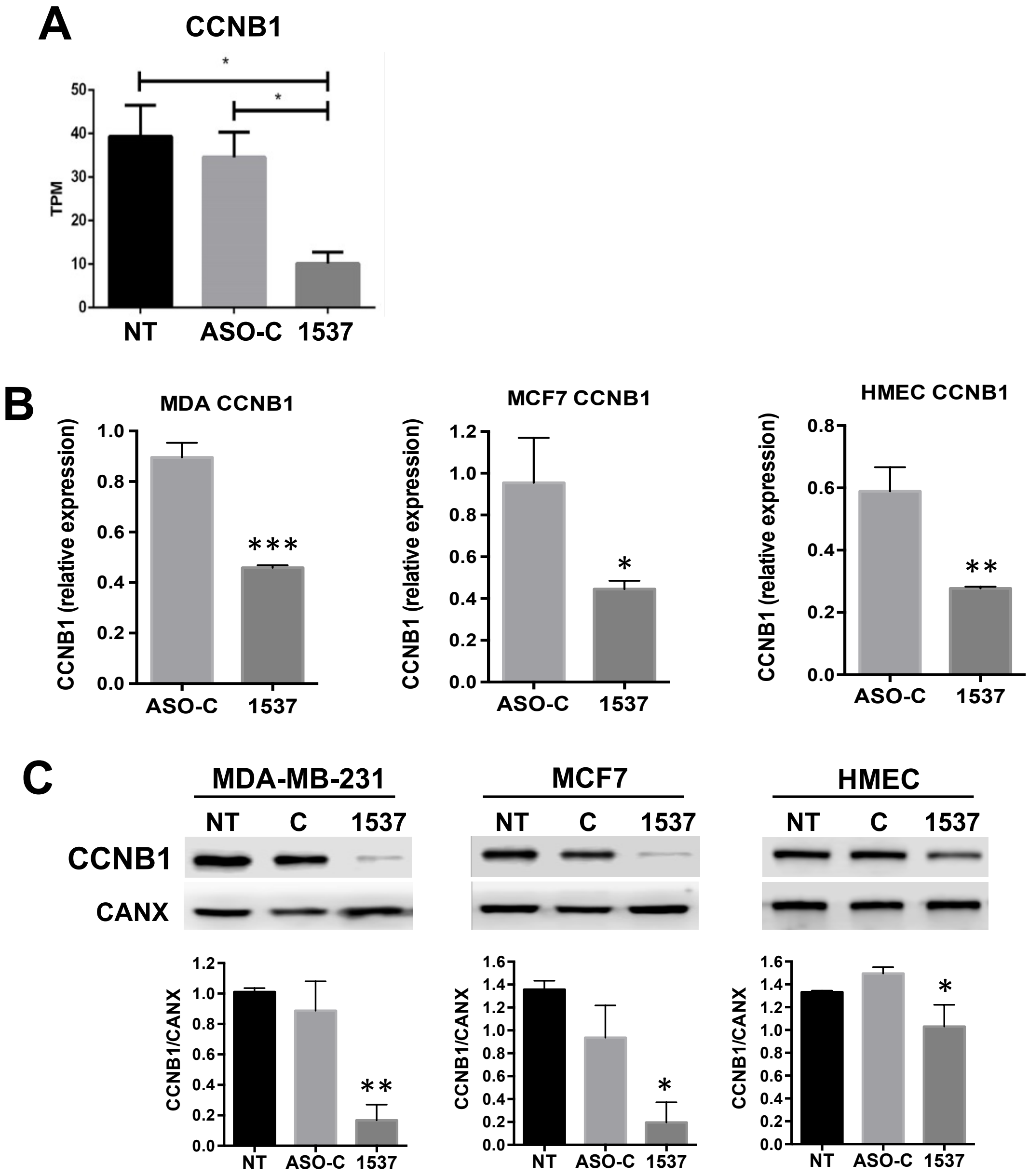

Figure S3: Changes in *CCNB1* expression after ASncmtRNA KD. **A**, Relative *CCNB1* levels in MDA-MB-231 cells after a 24 h treatment with Andes-1537, ASO-C or non-treated (NT), obtained from transcriptomic data and expressed in TPM. **B**, *CCNB1* mRNA levels are decreased 2-fold in MDA-MB-231, MCF7 and HMEC, as evidenced by RT-qPCR. Data is presented relative to NT cells. **C**, Cells treated as in B were subjected to Western blot analysis, which showed a reduction in *CCNB1* protein levels in all three cell types, but much more marked in both tumor cell lines compared to normal HMECs. Calnexin (*CANX*) was used as loading control. **p* < 0,05, ***p* < 0,01, ****p* < 0,001.
